# Comparative transcriptional profiling analysis of the effect of heat waves during embryo incubation on the hatchlings of the Chinese soft‐shelled turtle (*Pelodiscus sinensis*)

**DOI:** 10.1002/ece3.3850

**Published:** 2018-03-10

**Authors:** Wei Dang, Hongliang Lu, Qiong Wu, Yuan Gao, Qinqin Qi, Handong Fan

**Affiliations:** ^1^ Hangzhou Key Laboratory of Animal Adaptation and Evolution Hangzhou Key Laboratory of Ecosystem Protection and Restoration School of Life and Environmental Sciences Hangzhou Normal University Hangzhou China; ^2^ School of Food Science and Biotechnology Zhejiang Gongshang University Hangzhou China

**Keywords:** differentially expressed genes, hatchlings, heat treatment, immunity, *Pelodiscus sinensis*, RNA‐seq quantification analysis

## Abstract

Temperature is one of most the important environmental factors that affect the ontogenesis of organisms. In this study, we incubated Chinese soft‐shelled turtle eggs at 28°C (control temperature, C treatment), a temperature with a 16°C cold shock and a 36°C heat shock twice per week (S treatment) or a ramp‐programmed temperature of 29 ± 9°C (with 12 hr (+) and 12 hr (−) every day) (F treatment). The incubation period, hatching success, hatchling weight, and locomotor performance were significantly different between the controls and the different heat treatment groups. The pathogen challenge results illustrated that hatchlings from the S treatment group were more resistant to bacterial infection, whereas hatchlings from the F treatment group were more vulnerable. We used RNA‐seq quantification analysis to identify differentially expressed genes (DEGs) of hatchlings in the S treatment group. Based on the functional annotation results for the DEGs, 9 genes were chosen to verify the RNA‐seq results. The background expression of DEGs was also analyzed for the three treatments, as was the regulation of the pathogen challenge. The results showed that 8 DEGs were related to the immune response after pathogen challenge and that temperature was an important factor in differential regulation of the immunity pathways.

## 1 INTRODUCTION

Animals can adjust to gradual changes in ambient temperature by several mechanisms, including modifying gene expression and metabolic pathways as well as by changing their phenotypes to adapt the new conditions and shifting to new habitats (Huey & Kingsolver, 1989; Pounds et al., 2006; Thomas, Franco, & Hill, 2006). However, sudden changes in temperature can threaten the survival of animals. Extreme climate events are frequently reported in many countries with gradual warming trends, and these events will likely be more frequent and more intense in the future (IPCC 2014). Sudden extreme heat waves can induce death for animals with limited tolerance for heat (Bondarenco, Kortner, & Geiser, 2014; Vitali et al., 2015). Global climate change combined with heat waves catastrophically affects both marine and terrestrial ecosystems (Lejeusne, Chevaldonne, Pergent‐Martini, Boudouresque, & Perez, 2010). Widespread mortality and shifts in the distribution of species as well as declines in biodiversity appear with changes in the ecosystem. Empirical studies also indicated that the structures of specific ecosystems and host–parasite interactions would be affected by simulated heat waves (Landis, Kalbe, Reusch, & Roth, 2012; Sentis, Hemptinne, & Brodeur, 2013; Smale, Yunnie, Vance, & Widdicombe, 2015).

Animals can choose the most suitable temperature for their development, and temperature can modify their performance and life history traits as well (Angilletta, Sears, & Pringle, 2009; Parker & Andrews, 2007). Unpredictable extreme temperatures mean that organisms must respond rapidly for protection. The process is energetically costly, with the impairment of enzymatic function, membrane structure, and oxygen supply (Leicht, Jokela, & Seppala, 2013). Research based on experiments that were designed with simulated heat waves demonstrated that the survival rate, morphology, growth, and reproduction could be affected, and the levels of defense traits were ultimately reduced (Bauchinger, McWilliams, & Pinshow, 2011; Leicht et al., 2013). In *Drosophila*, genetic perturbation occurred during the spring 2011 heat wave in Western Europe, during which the genetic constitution of the population transiently shifted to summer‐like frequencies (Rodriguez‐Trelles, Tarrio, & Santos, 2013).

In ectotherms, the development and efficiency of the immune system, which is susceptible to the external temperature and is one of the key systems for survival, were markedly affected by temperature changes (Rijkers, Frederix‐Wolters, & Van Muiswinkel, 1980; Truscott & White, 1990). The expression of immune parameters, which are related to innate and acquired immune functions, is sensitive and responds quickly along with changes in phenotypes. Genes related to the immune system, such as heat‐shock proteins (Hsps), superoxide dismutase (SOD), and tumor necrosis factor (TNF), have been selected for gene expression profiling (Dittmar, Janssen, Kuske, Kurtz, & Scharsack, 2013; Zhang et al., 2014). Despite the growing appreciation of the effects of heat waves on organisms, research about ectotherms is rare. Recently reported results about ectotherms have mostly addressed the effects of heat waves on the phenotypes of animals, whereas molecular mechanisms and gene adaptations were rarely considered.

In this study, we used the Chinese soft‐shelled turtle, *Pelodiscus sinensis*, which is genetically sex determined (GSD) (Ji, Chen, Du, & Chen, 2003). Therefore, we designed this experiment without regard to sex. Most of the research about Chinese soft‐shelled turtle embryo incubation has been conducted at a constant temperature in the laboratory. The constant incubation temperature range of the Chinese soft‐shelled turtle is 24–34°C, and temperature extremes will cause stress, which would lead to deformities and to disruptions of embryonic development (Du & Ji, 2003). In the wild, ectotherms experience temperature fluctuation, but there are few studies addressing the effects of extreme temperature changes. In this study, we used two experimental incubation temperatures with fluctuations to mimic the natural extreme temperatures with climate change, and the lowest and highest temperatures were outside the range of constant temperatures used for incubation. Morphological parameters of hatchlings were recorded and analyzed. We used a pathogen isolated from a diseased Chinese soft‐shelled turtle to challenge the hatchlings and evaluated the immunity of hatchlings from different treatments. The effects of temperature on phenotypes of ectotherms have been widely reported, but the underlying molecular mechanisms remain unclear. In this study, we intended to identify the genes responsive to heat stress, especially genes related to immunity and other pathways. RNA‐seq quantification analysis is one of the RNA deep‐sequencing technologies for genome‐wide transcriptome analysis (Wang, Gerstein, & Snyder, 2009). We used RNA‐seq quantification analysis to determine the complexity of gene expression regulation affected by heat waves. The gene data mining is the first step to illustrate the thermal adaptation of ectotherms and is also important for identifying the problematic effects of global climate change.

## 2 MATERIAL AND METHODS

### 2.1 Study species and pathogen strain

The Chinese soft‐shelled turtle is one of the main aquaculture species in China and is distributed across China and Southeastern Asia. We collected 633 freshly laid fertilized eggs, which were identified by a white patch on the shell surface, from a farm located in Hangzhou, Zhejiang, in July 2014. The mean clutch size of the female turtles is 18, and the mean egg mass was determined to be 4.19 ± 0.58 g using an electronic balance (Mettler Toledo AB135‐S, Germany). The pathogen used with the Chinese soft‐shelled turtles was *Aeromonas hydrophila* TL1, which was isolated from the liver of a diseased turtle. *A. hydrophila* TL1 was cultured in Luria‐Bertani (LB) liquid medium at 28°C.

### 2.2 Egg incubation and hatching rate

**Figure 1:**
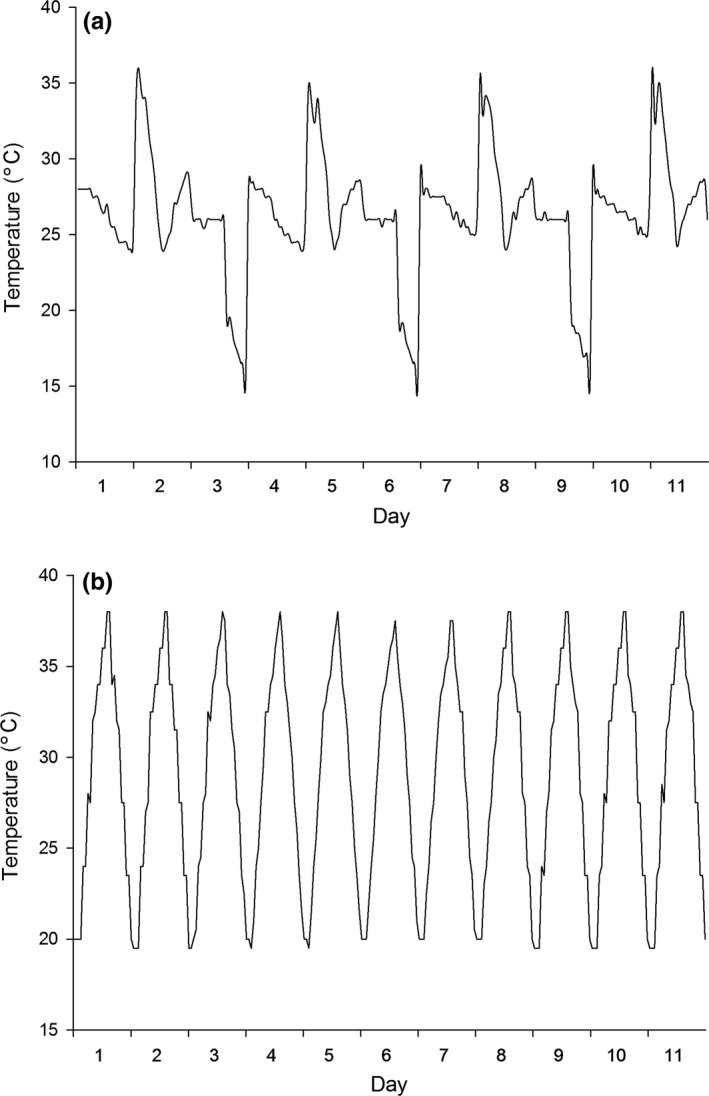
The two heat wave treatments of the Chinese soft‐shelled turtle eggs. (a) The S treatment, an incubation temperature with a 16°C cold shock and a 36°C heat shock twice per week; (b) The F treatment, a ramp‐programmed temperature of 29 ± 9°C with 12 hr (+) and 12 hr (−) every day

The eggs were placed in plastic containers filled with moist vermiculite at −220 kPa (1 g water/1 g vermiculite). Each container contained eight eggs. There were three types of temperature settings for incubators (Ningbo Life Science and Technology Ltd., China). The control temperature was 28°C (the C treatment), which is the average land surface temperature from May to July to in Hangzhou over 10 years and the most suitable temperature for incubation of Chinese soft‐shell turtles. The first experimental temperature condition was a temperature with a 16°C cold shock and a 36°C heat shock twice per week (the S treatment, Figure 1a), and the second was a ramp‐programmed temperature of 29 ± 9°C, with 12 hr above 29°C (+) and 12 hr below 29°C (−) every day (the F treatment, Figure 1b). The eggs were randomly distributed into three incubators to minimize maternal effects. The containers in incubators were examined twice per week to promptly remove the eggs in which embryogenesis had terminated and to keep the vermiculite at the appropriate moisture level. The containers were moved to different shelves according to a predetermined schedule to minimize any effects of thermal gradients inside the incubators.

### 2.3 Incubation periods and hatching success

Toward the end of incubation, the containers were monitored once per day for newly emerging hatchlings. The incubation periods recorded for the day lasted from the beginning of incubation to the emergence of the hatchlings, and the hatching rates are expressed as the percentage of eggs that hatched live turtles at the end of the experiment. After emergence, the hatchlings were maintained in the containers until the yolk was entirely absorbed, after which they were kept in a temperature‐controlled room at 28 ± 1°C with a 12 hr light/12 hr dark cycle.

### 2.4 Hatchling traits and locomotor performance

After the yolk had been entirely absorbed, the weights of hatchlings were measured with an electronic balance (Mettler Toledo AB135‐S, Germany) to the nearest 1 mg. The lengths, widths, and heights of the turtle shells were recorded with digital Vernier calipers (Mitutoyo). Then, each hatchling was chased along a 1.2‐m racetrack filled with water at a depth of 40 mm and at a constant temperature of 28°C. Swimming performance was recorded with a Panasonic video camera. Videotapes were later analyzed for sprint speed in the fastest 30‐mm interval.

### 2.5 Experimental infection and virulence analysis

We used the median lethal dose (LD50) for hatchlings after pathogen infection to determine the immunocompetence of the turtles from different incubation temperatures. Briefly, the hatchlings from each incubation temperature were divided into four groups, each containing 20 hatchlings. The four groups were separately challenged with 5 × 10^3^ colony‐forming units (CFU), 5 × 10^4^ CFU, 5 × 10^5^ CFU, or 5 × 10^6^ CFU of TL1. The turtles were monitored for 10 days, and the mortalities were recorded every day.

### 2.6 RNA‐seq library preparation and sequencing

Based on the bacterial infection test results, we chose hatchlings from the control and the S treatment groups for analysis of differentially expressed genes. From each group, three hatchlings were randomly selected before immune challenge, and the livers of those hatchlings were removed aseptically and frozen in liquid nitrogen. Total RNA was extracted using TRIzol (Invitrogen, Carlsbad, CA, USA) and digested with DNase I (NEB, Ipswich, MA, USA). Poly‐(A) mRNA was purified using a Dynabeads mRNA purification kit (Invitrogen, Carlsbad, CA, USA) and fragmented into small pieces (approximately 200 bp) with first‐strand buffer (Invitrogen, Carlsbad, CA, USA). The first‐strand cDNA was synthesized using random hexamer primers, First‐Strand Master Mix, and Super Script II reverse transcriptase (Invitrogen, Carlsbad, CA, USA). The second‐strand cDNA was synthesized with Second‐Strand Master Mix (Invitrogen, Carlsbad, CA, USA). The double‐stranded cDNA was purified with Agencourt AMPure XP beads (Beckman Coulter, Brea, CA, USA), and barcoded adapters were then ligated to the end of the cDNA fragments. The products were selected and enriched by PCR amplification. Qualified library products were sequenced via the Ion Proton platform at BGI Shenzhen.

### 2.7 Transcriptome data processing and analysis of differential gene expression

The original image data collected from the Ion Proton platform were converted into sequence data. The adaptor sequences and low‐quality reads were filtered to obtain high‐quality clean reads. After data quality statistics, clean reads were mapped against the genome and genes of *P. sinensis*, which are available at http://www.ncbi.nlm.nih.gov, by the Torrent Mapping Alignment Program (TMAP). The gene expression levels were quantified with the *Sailfish* software package. The RPKM method, which calculates the reads per kilobase per million mapped reads (RPKM = total exon reads/mapped reads in million X exon length in kb), was used for calculating the expression level. The Noiseq package method (Tarazona, García‐Alcalde, Dopazo, Ferrer, & Conesa, 2011) was used to screen differentially expressed genes (DEGs) between two groups. The data were log2 transformed to identify the genes with significant differential expression during heat stress [log2‐fold change (FC) ≥1 or log2 FC ≤−1 and *p* value < .05]. Gene ontology analysis was used to identify the main biological functions performed by the DEGs. The calculated *p*‐value underwent a Bonferroni Correction (Abdi, 2007), and a corrected *p*‐value ≤ .05 was used as a threshold. Reads showing significant homology against Chinese soft‐shelled turtle genes were mapped onto metabolic pathways using the KEGG database (http://www.genome.jp/kegg/pathway.html).

### 2.8 Changes in DEG expression after heat treatment and bacterial infection

According to the analysis using the KEGG database, we designed primers for 9 DEGs; these primers are shown in Table S1. At the same time, we also examined the changes in expression of these genes at 12 hr and 48 hr after bacterial infection. Hatchlings (5/group) from the three treatments without bacterial challenge and from the 5 × 10^5 ^CFU group at 12 hr and 48 hr postchallenge were sacrificed. Livers were removed and frozen in liquid nitrogen. Total RNA was extracted using TRIzol (Invitrogen, CA, USA), and the first‐strand cDNA was obtained using the PrimeScript^™^ RT Reagent Kit with gDNA Eraser (Perfect Real Time) (Takara, Dalian, China). The designed primers were confirmed to be specific through PCR and sequencing. Then, quantitative real‐time PCR was conducted according to a previously described protocol (Dang, Lu, et al., 2015; Dang, Zhang, & Du, 2015). The specific qRT‐PCR products were verified by electrophoresis.

### 2.9 Data analysis

The software packages Statistica 6.0 (StatSoft, Tulsa, USA) and SPSS 13.0 were used for data analysis. A Kolmogorov–Smirnov test was used for normality of distributions, and Bartlett's test was used for homogeneity of variances. Analysis of variance (ANOVA) was used to determine the influence of incubation temperature on the incubation period, and analysis of covariance (ANCOVA) was used to determine the influence of incubation temperature on hatchling mass, with the initial egg mass as the covariate. When the hatchlings were exposed to bacterial infections, a *G* test was used to analyze the among‐treatment differences in mortality, and a probit model was used to determine the median lethal dose (LD50). Values are presented as the mean ± *SE* and range, and the significance level was set at *p *=* *.05 throughout this study.

## 3 RESULTS

### 3.1 Incubation period and hatching rate

There were significant differences among the treatments for incubation period (*F*_2, 433_ = 3100.2, *p *<* *.0001). Hatchlings from the S treatment possessed the longest incubation period and highest hatching success (76.78%). Both the incubation period and hatching success (71.09%) of the C treatment hatchlings were intermediate. Hatchlings from the F treatment possessed the shortest incubation period and lowest hatching rate (58.77%). The specific data are all shown in Table 1.

**Table 1:** Eggs used for the different treatment, incubation period, and hatching rate from different treatments

Incubation temperature (**°**C)	Egg weight (g)	Incubation period (days)	Hatching rate (%)
28 (C treatment)	4.154 ± 0.047	53.8 ± 0.1	71.09
16–36 (S treatment)	4.249 ± 0.045	63.6 ± 0.1	76.78
20–38 (F treatment)	4.160 ± 0.052	53.3 ± 0.1	58.77

### 3.2 Hatchling traits and locomotor performance

The weights of hatchlings from the F treatment, which were the lightest, were significantly different from those in the other two treatments (*F*_2, 433_ = 11.77, *p *<* *.001). There were no significant differences among treatments in terms of shell length (*F*_2, 433_ = 0.55, *p *=* *.575), shell width (*F*_2, 433_ = 1.71, *p *=* *.182), and body height (*F*_2, 433_ = 2.05, *p *=* *.130). The hatchlings from the F treatment showed the fastest swimming speed, which was significantly faster than in the other two treatments (*F*_2, 55_ = 8.88, *p *<* *.001), whereas there were no significant differences between hatchlings from the C and S treatments. The specific data are all shown in Table 2.

**Table 2:** Hatchling traits and locomotor performance from different treatments

Incubation temperature	Hatchling weight (g)	Shell length (mm)	Shell width (mm)	Shell height (mm)	Swimming speed (m/s)
28 (C treatment)	2.934 ± 0.038	24.892 ± 0.135	22.950 ± 0.122	8.258 ± 0.074	0.132 ± 0.010
16–38 (S treatment)	2.963 ± 0.039	25.059 ± 0.130	22.840 ± 0.117	8.371 ± 0.071	0.137 ± 0.009
20–38 (F treatment)	2.801 ± 0.039	25.076 ± 0.149	22.619 ± 0.133	8.480 ± 0.081	0.183 ± 0.009

### 3.3 Immunity

**Figure 2:**
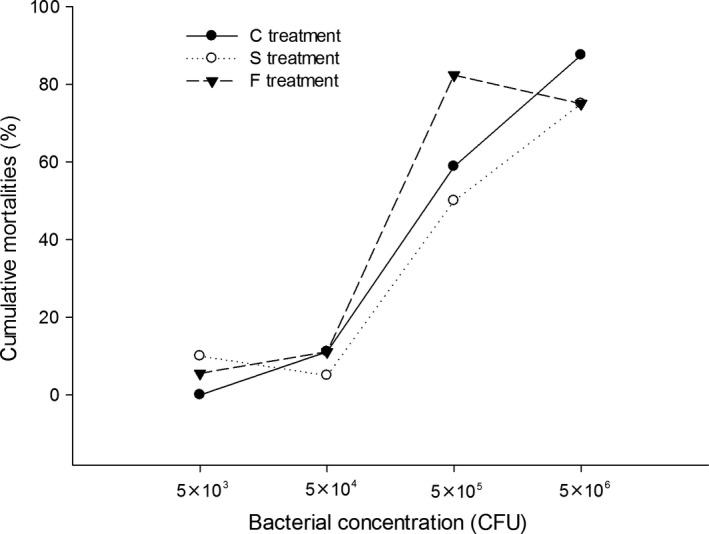
The cumulative mortalities of Chinese soft‐shelled turtle hatchlings from three different temperature treatments. The hatchlings were infected with various concentrations (5 × 10^3^ colony‐forming units (CFU), 5 × 10^4^ CFU, 5 × 10^5^ CFU, or 5 × 10^6^ CFU) of *Aeromonas hydrophila* TL1 and monitored daily for mortality. The cumulative mortality was calculated at the end of the monitoring period

There were no significant differences in cumulative mortality under different temperature treatments at challenges with 5 × 10^3 ^CFU (*G *=* *2.67, *df* = 2, *p *=* *.263), 5 × 10^4^ CFU (*G *=* *0.64, *df* = 2, *p *=* *.725), and 5 × 10^5^ CFU (*G *=* *2.20, *df* = 2, *p *=* *.332). At the higher challenge concentration of 5 × 10^6^ CFU (*G *=* *6.54, *df* = 2, *p *=* *.038), the cumulative mortality in the S treatment was lower than those in the C and F treatments (Figure 2). Hatchlings from the F treatment had the lowest median lethal dose (LD50) of the pathogen (3.32 × 10^5^), followed by hatchlings from the C treatment (7.44 × 10^5^); hatchlings from the S treatment possessed the highest LD50 (1.29 × 10^6^).

### 3.4 RNA‐seq mapping and analysis of differentially expressed genes

The data analyzed in this study were submitted to the NCBI Sequence Read Archive under accession no. SRP104379. We sequenced three samples from each group, and for each sample, we collected more than 12 million clean reads. Clean reads were mapped to a reference genome and to reference genes at mapping rates that were higher than 97% and 58%, respectively. There were more than 18,000 expressed genes for each sample. The specific number of clean reads yielded from each sample, the genome and gene mapping rates, and the expressed genes are shown in Table S2. As there were three replicates per group, the RPKM values of each gene in each replicate showed slight differences. Based on the different DEG screening methods, we obtained various statistics about the DEGs, which are shown in Table 3. In this study, we obtained 84 genes that were differentially expressed between two groups. These significantly upregulated or downregulated genes are listed in Table S3.

**Table 3:** Statistics of differentially expressed genes

Pairs/type	DiffGene (up)	DiffGene (down)
C vs. S	57	27
C1 vs. S1	2,333	1,891
C2 vs. S2	2,105	2,114
C3 vs. S3	2,186	1,657

### 3.5 Functional annotation of differentially expressed genes

The 84 DEGs were mapped to GO and KEGG ontology (KO) terms to assign gene functions. Among the component ontology terms, most DEGs were associated with cells, cell parts, and organelles. Among the function ontology terms, most DEGs were related to binding and catalytic activity. Among the process ontology terms, most DEGs were involved in muscle‐associated processes and metabolism (Table S4). Seventy‐three DEGs with pathway annotations were identified. Because there were no available previous reports of pathways for the Chinese soft‐shelled turtle or other reptiles, the annotations mostly used human pathways as references. Most of the enriched pathways were involved in disease or infection (39.73%), signaling pathways (13.70%), and metabolism (10.96%) (Table S5). Most of the DEGs involved in signaling pathways were related to disease or infection.

### 3.6 Effects of heat treatment and bacterial challenge

**Figure 3:**
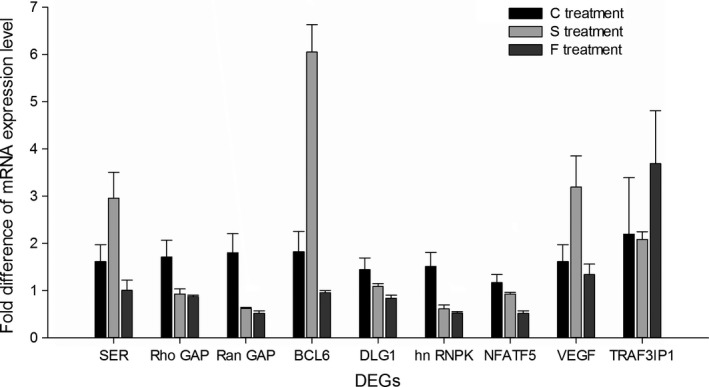
The expression of the 9 selected DEGs in liver of Chinese soft‐shelled turtle hatchlings from the three temperature treatments. The expression levels of 9 DEGs in liver were determined by quantitative real‐time reverse‐transcription PCR and normalized to those of β‐actin mRNA. Levels in the C treatment were set as 1. Vertical bars indicate the means ± *SE* (*N* = 5)

**Figure 4:**
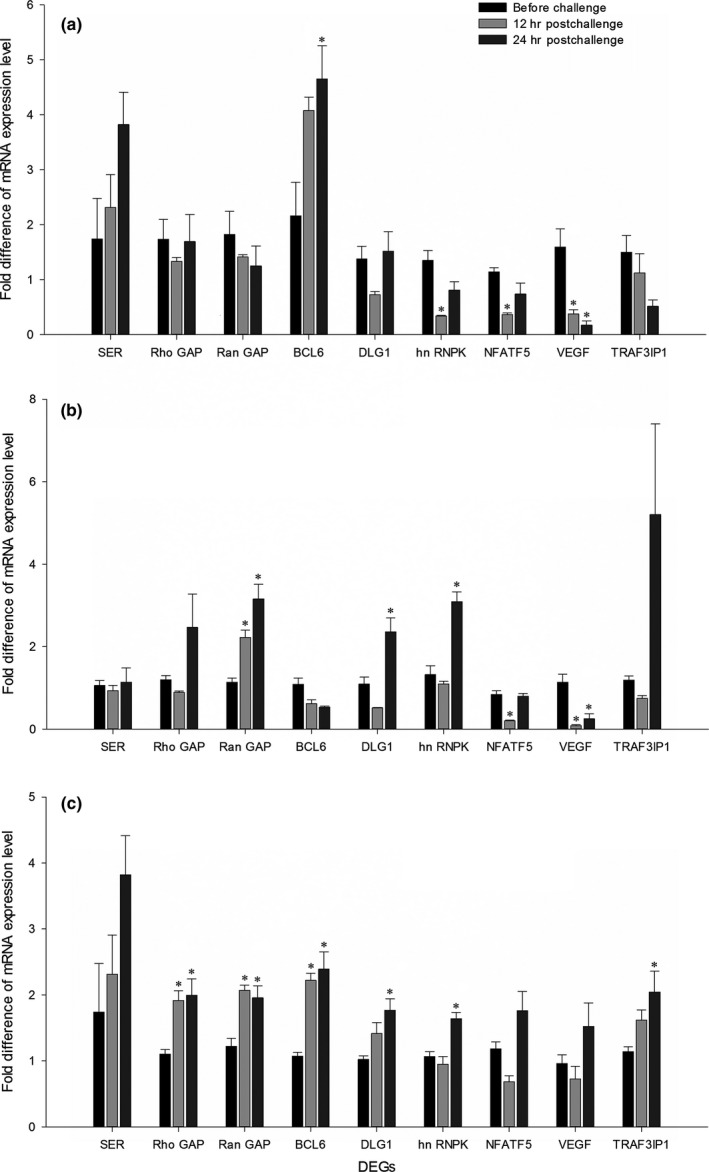
Changes in expression of 9 DEGs in Chinese soft‐shelled turtle hatchlings from the three temperature treatments at 12 hr and 48 hr after pathogen challenge. (a) The C treatment; (b) The S treatment; (c) The F treatment. The expression levels of 9 DEGs in liver of different treatment hatchlings were determined by quantitative real‐time reverse‐transcription PCR and normalized to those of β‐actin mRNA. Values are shown as the means ± *SE* (*N* = 5). Significant differences between hatchlings without pathogen challenge and hatchlings at 12 hr and 48 hr postchallenge are indicated with asterisks. *p < .05

We chose 9 DEGs to verify the results of the RNA‐seq and analyze the gene expression among the three temperature treatments by qRT‐PCR. The results showed that the expression patterns of 6 of the 9 DEGs were identical to the patterns determined by RNA‐seq (Figure 3). For the same 9 DEGs, we also analyzed the expression patterns in the S treatment, and we found that the expression patterns were not simply opposite between the F treatment and the S treatment and that some DEGs showed similar expression patterns in the two groups (Figure 3). As we could not simply determine whether these 9 DEGs were related to the immunity of hatchlings, we examined the changes in expression of these 9 DEGs after bacterial challenge in the different treatment groups. The expression patterns of each gene were different in different treatment groups. After bacterial challenge, BCL1 was observed to be upregulated in the C and F treatments but not in the S treatment. Also after bacterial challenge, NFATF5 and VEGF were found to be downregulated in the C and S treatments but not in the F treatment. In the F and S treatments, we found that three genes, RanGAP, DLG1, and hnRNPK, showed the same expression pattern, namely, upregulation after bacterial challenge, whereas these genes were not differentially expressed in the C treatment (Figure 4).

## 4 DISCUSSION

### 4.1 Temperature and parameters

Temperature plays an important role in the life histories of organisms. As weather conditions with more frequent extreme temperatures appear, research about ectotherms in the laboratory is shifting from constant temperature to fluctuating temperatures. In a previous study about Chinese soft‐shelled turtle (Du & Ji, 2003), turtles under a lower incubation temperature underwent a longer incubation period. The hatching success was low at the lowest (23°C) and highest (34°C) temperatures. The body mass was not affected under constant temperatures. Ji et al. incubated the Chinese soft‐shelled turtle under fluctuating temperatures with different magnitudes of fluctuation (30 ± 3°C and 30 ± 5°C) (Li, Zhou, Wu, Wu, & Ji, 2013). For the turtles at 30 ± 5°C, the incubation period was longer, the hatching success was lower, and hatchling size was smaller, but the swimming speed was not affected. In other studies about diel temperature fluctuations during reptile incubation, incubation period increased or decreased depending on the mean incubation temperature and thermal flux, and lower rates of hatching success resulted from the fluctuations exceeding the constant optimal developmental range (Ashmorengi & Janzen, 2003; Les, Paitz, & Bowden, 2009; Neuwald & Valenzuela, 2011; Warner & Shine, 2011). In red‐eared slider turtle (*Trachemys scripta*), Bowden et al. alternated the fluctuation frequency of incubation temperature with 12‐hr, 24‐hr, and 48‐hr cycles; the hatching success was not affected, and the 48‐hr cycle treatment showed a significantly longer incubation period, ~1 day longer than in the other two treatments (Bowden et al., 2014). The experimental temperatures for the red‐eared slider turtle were within the optimal developmental range. In this study, we mimicked the natural temperature fluctuation and designed two different heat waves to illustrate the effects of temperature, and the lowest and highest temperatures of these two fluctuating treatments were outside the range of 24–34°C (Du & Ji, 2003), which is the constant incubation temperature range for the Chinese soft‐shelled turtle. The mean temperatures of the two heat wave treatments were 26°C (S treatment) and 29°C (F treatment). Although the temperatures in the S treatment were outside the optimal development range for Chinese soft‐shelled turtles, the hatchlings in the S treatment showed a longer incubation period and higher hatching success. We cannot currently determine whether the mean temperature or the fluctuation frequency induced these results.

In reptiles, incubation temperature can have many effects on posthatching traits, including fitness. Immunity, providing a generalized defense against infection, is one important component of fitness in vertebrates. In Chinese soft‐shelled turtle and map turtle hatchlings, stronger immune responses were shown after incubation under a lower constant temperature (24°C) (Dang, Lu, et al., 2015; Dang, Zhang, et al., 2015; Freedberg et al., 2008). In painted turtle, hatchlings from the fluctuating treatments mounted a greater swelling response to an intraperitoneal priming injection of phytohemagglutinin than did hatchlings from the constant treatments (Les et al., 2009). In this study, the bacterial challenge results showed that fluctuations in incubation temperature definitely affected the turtle mortality after bacterial challenge. As described above, the mean temperature and the magnitude and frequency of the fluctuations in the incubation temperature should be all important considerations. In the future, we suggest refining the temperature design to test the effects of these factors separately. Here, we focused on the underlying molecular mechanism associated with immunity and temperature, and the DEGs were examined first.

### 4.2 DEGs associated with heat and bacterial challenge

Based on the functional annotation of DEGs in the KEGG ontology database, 73 DEGs were annotated, and 29 DEGs were related to disease or infection. There were 10 DEGs participating in signaling pathways, and almost all these DEGs are also involved in disease or infection. There were 8 DEGs that participate in nutrient substance metabolism. Most DEGs are not studied in ectotherms. The 9 DEGs selected for further analysis were all involved in pathways associated with disease or infection, and they are associated with signal transduction. Sarcoplasmic/endoplasmic reticulum calcium ATPase 1 (SERCA1) is associated with muscle development. Two GTPase‐activating proteins (GAPs) are broadly related to signal transduction in multiple cell types. Heterogeneous nuclear ribonucleoprotein K (hnRNP‐K) is a positive regulator of vascular endothelial growth factor A (VEGF‐A), which is related to stress. The other four genes, B‐cell lymphoma 6 protein (BCL6), nuclear factor of activated T cells 5 (NFAT5), disks large homolog 1 (DLG1), and TRAP‐3 interacting protein 1 (TRAF3IP1), are involved in the immune response in B cells and T cells.

Research about SERCA1 in humans has shown that this protein is one of the most important contributors to removing calcium from the cytoplasm in skeletal muscle (Guglielmi et al., 2016). Defective function of SERCA1 protein was associated with a type of myopathy and with loss of muscle mass (Mázala et al., 2015). The results as verified by qRT‐PCR were not consistent with the RNA‐seq results. The expression of SERCA1 was slightly but nonsignificantly upregulated in the different temperature treatments and after pathogen challenge. In this study, we speculated that SERCA1 was not related to long‐term temperature treatment and immunity in the Chinese soft‐shelled turtle. The RNA‐seq analysis also identified other DEGs, such as Myosins, that are involved in muscle development, but these genes were not verified here. A new question is whether the heat stress affected muscle development, which would merit further investigation.

Two GTPase‐activating proteins (GAPs), Rho GTPase‐activating protein 5 (Rho GAP), and Ran‐specific GTPase‐activating protein (Ran GAP) were found to be differentially expressed after heat treatment. The verification results showed that the expression of Rho GAP was slightly downregulated, which was not consistent with the RNA‐seq result, and the expression of Ran GAP was significantly upregulated after heat treatment. GAPs are common in *H*uman and Drosophila, and they inactivate typical Ras superfamily members, such as Rho and Ran, by accelerating the low intrinsic rate of GTP hydrolysis (Bernards, 2003). Rho GTPases are found in various kinds of cells; *work as important* intracellular signaling switches; are involved in actin polymerization, focal adhesion complex assembly and function, cell cycle progression, membrane trafficking, cell adhesion, and cell polarity; and are reported to be associated with diseases such as cancer and neurological and cardiovascular disorders (McKerracher, Ferraro, & Fournier, 2012; Peck, Douglas, Wu, & Burbelo, 2002). As negative regulators of Rho GTPases, knockdown or overexpression of Rho GAPs was reported to result in morphological changes in tumor cells (Saito, Ozawa, Hibino, & Ohta, 2012). Ran GTPases participate in various nuclear events, and mutation of Ran GAP caused defects in RNA processing and nucleocytoplasmic mRNA transport in yeast (Bischoff, Krebber, Kempf, Hermes, & Ponstingl, 1995). Upregulated expression of Rho GAP and Rho GAP after pathogen challenge was only found in the heat treatment group*,* not in the control group. These results may be related to the higher background expression of these two genes in the control group.

hnRNP‐K is a component of the ribonucleoprotein particles, serves as a nucleic acid‐linking docking platform, and is involved in the regulation of chromatin structure, transcription, pre‐mRNA *proce*ssing, splicing, mature mRNA transport to the cytoplasm, translation, nuclear transport, signal transduction, and DNA repair (Gao et al., 2013). hnRNP‐K was found to bind to elongation factor 1α and to the promoter of VEGF (Bomsztyk, Van Seuningen, Suzuki, Denisenko, & Ostrowski, 1997; Uribe, Guo, Shin, & Sun, 2011). VEGF‐A was originally discovered to function in vasculogenesis and angiogenesis and participates in preventing apoptosis in neurons and in promoting their proliferation and survival (Zhang, Bao, Hambly, & Gillies, 2009). More recently, VEGF‐A has been found to be produced in response to stressors, such as hypoxia, glucose deprivation, and extracellular pH (Latham, Molina‐Paris, Homer‐Vanniasinkam, & Ponnambalam, 2010). VEGF‐A was found to be associated with disease development and has even been proposed to function as a tumor biomarker (Latham et al., 2010). After the pathogen challenge, we found similar expression patterns for hnRNP‐K and VEGF‐A.

BCL6 is a transcriptional repressor participating in the germinal center (GC) reaction of mature B cells and CD4^+^ T‐follicular helper cells (Basso & Dalla‐Favera, 2012; Bunting & Melnick, 2013). In the presence of pathogens, the expression of BCL6 was significantly upregulated, except in the S treatment, in which the background expression level of BCL6 was significantly higher than in the other two treatments. NFAT proteins are nuclear transcription factors that were first found in activated T cells, and they regulate various cytokines involved in the immune system (De Boer, Mordvinov, Thomas, & Sanderson, 1999). Unlike the other four members of the NFAT family, which are regulated by calcium–calcineurin, NFAT5 is found in all cell types and is regulated by osmotic stress (Macian, 2005). DLG1 regulates Ag‐dependent cytoskeletal and signaling events by localizing to the junction between T cells and antigen‐presenting cells, also known as the immunological synapse (Crocetti, Silva, Humphries, Tibbs, & Miceli, 2014; Zanin‐Zhorov et al., 2012). Tumor necrosis factor receptor‐associated factor 3 (TRAF3) is an adaptor molecule that links upstream receptor signals to downstream gene activation in both adaptive immune cells and the innate immune system (Oganesyan et al., 2006; Yi, Lin, Stunz, & Bishop, 2014). TRAF3IP1 interacts with TRAF3 and is also important in the regulation of the immune response (Cheng et al., 2016). TRAF3IP1 was also found to bind both actin and tubulin to regulate cytoskeletal dynamics (Berbari et al., 2011). In this study, we found that NFAT5 was downregulated both in the heat treatment and after pathogen challenge, but the other genes related to immunity cells were upregulated after pathogen challenge in the groups subjected to heat treatment.

These 9 selected DEGs did not all show significant changes in expression in either temperature treatment. Temperature must be an important factor affecting gene expression pathways. The environmental temperature affects the entire physiology of ectotherms, and genes are the fundamental inherited factors that determine animal development. As the global environment deteriorates, population decreases or even the extinction of these animals seems likely. We attempted to identify the underlying effect of the environmental change in order to protect these animals.

Heat‐shock proteins are also associated with changes in temperature. In loggerhead sea turtle, the expression of genes in the *heat‐shock* protein family was upregulated after heat‐shock treatment (Bentley, Haas, Tedeschi, & Berry, 2017), and in invertebrates, heat‐shock proteins were found to be differentially expressed after heat treatment (DeSalvo et al., 2008; Gleason & Burton, 2015; Huang et al., 2017). However, we did not observe differential expression of heat‐shock proteins in Chinese soft‐shelled turtles. In our studies, the embryos completed development during the heat treatment, and we later analyzed the difference in just one organ. In our previous studies about the Chinese soft‐shelled turtle, the different constant incubation temperatures did not affect the expression of Hsp70 in liver of the hatchlings (Dang, Lu, et al., 2015; Dang, Zhang, et al., 2015). Further, the heat treatments in the other studies were short. We suggest that the embryos of Chinese soft‐shelled turtles acclimated to the incubation temperatures, and the expression of heat‐shock proteins returned to normal levels because constant high expression levels would be at the cost of other protein functions.

In conclusion, we incubated the Chinese soft‐shelled turtle eggs in two heat wave conditions and used 28°C as the control temperature. The incubation period, hatching success, hatchling weight, and locomotor performance were significantly different between the control group and the different heat wave treatment groups. The bacterial challenge results demonstrated that hatchlings from the group treated with two heat shocks during incubation (S treatment) were more resistant to bacterial infection. We used RNA‐seq quantification analysis to identify differentially expressed genes in hatchlings in the S treatment group. The functional annotation of DEGs with the KEGG ontology database implied that they include genes related to immunity and metabolism. The genes reported in this study will provide a fundamental basis for discovering novel genes in the responses of Chinese soft‐shelled turtles to different temperatures.

## CONFLICT OF INTEREST

None declared.

## AUTHOR CONTRIBUTIONS

Wei Dang and Hongliang Lu designed the experiment, analyzed results, and wrote this paper. Qiong Wu, Yuan Gao, Qinqin Qi, and Han Dongfan conducted the experiment.
